# Functional Design and Clinical Implications of Modern Soccer Footwear: A Comprehensive Narrative Review

**DOI:** 10.3390/jfmk11010062

**Published:** 2026-01-30

**Authors:** Andrea Demeco, Nicola Marotta, Marco Megna, Andrea Racinelli, Bruno Pansera, Antonio Frizziero, Ilona Yosypchuk, Stefano Palermi, Marco Vecchiato, Ennio Lopresti, Alessandro de Sire, Antonio Ammendolia

**Affiliations:** 1 Physical and Rehabilitative Medicine, Department of Medical and Surgical Sciences, University of Catanzaro “Magna Graecia”, 88100 Catanzaro, Italy; marco.megna001@studenti.unicz.it (M.M.); andrea.racinelli@studenti.unicz.it (A.R.); bruno.pansera002@studenti.unicz.it (B.P.); annailona97@libero.it (I.Y.); ennio.lopresti@studenti.unicz.it (E.L.); alessandro.desire@unicz.it (A.d.S.); ammendolia@unicz.it (A.A.); 2 Research Center on Musculoskeletal Health, MusculoSkeletalHealth@UMG, University of Catanzaro “Magna Graecia”, 88100 Catanzaro, Italy; nicola.marotta@unicz.it; 3 Physical and Rehabilitative Medicine, Department of Experimental and Clinical Medicine, University of Catanzaro “Magna Graecia”, 88100 Catanzaro, Italy; 4 Department of Rehabilitation, Azienda Socio Sanitaria Territoriale (ASST) Gaetano Pini-Centro Specialistico Ortopedico Traumatologico (CTO), Piazza Cardinal Ferrari 1, 20122 Milan, Italy; antonio.frizziero@unimi.it; 5 Departmental Faculty of Medicine and Surgery, UniCamillus-Saint Camillus International University of Health Sciences, 00187 Rome, Italy; stefano.palermi@unicamillus.org; 6 Sports and Exercise Medicine Division, Department of Medicine, University of Padova, 35122 Padova, Italy; marcovecchiato.md@gmail.com

**Keywords:** football, footwear, biomechanics, injury, performance, injuries, sports

## Abstract

Soccer is the most widely practiced sport globally, but is also associated with a high incidence of lower limb injuries. Among multiple risk factors, soccer footwear represents a crucial biomechanical interface affecting traction, proprioception, and joint loading. This narrative review aims to explore how each component of modern soccer footwear impacts performance and injury risk, with a focus on evidence-based functional customization. A comprehensive narrative review of available literature was conducted across PubMed, Scopus, and Web of Science, integrating biomechanical, clinical, and materials science studies. We included studies concerning the structures composing soccer technical footwear. Conical studs were associated with reduced rotational stiffness and lower joint torque, while bladed studs enhanced linear traction but increased ACL strain risk. Upper materials, such as knitted fabrics and engineered mesh, improve proprioception and thermal regulation but show trade-offs in durability and protection. Soleplate stiffness influenced load distribution and performance: increased stiffness improves sprinting but compromises multidirectional agility. Fatigue and proprioception were modulated by insole and soleplate synergy. Soccer footwear should be seen as a clinical and performance tool requiring evidence-based customization. Advances in material technology, 4D foot scanning, and plantar pressure mapping enable functional matching between footwear and athlete characteristics. Translating these insights into player-specific footwear designs may reduce injury rates and enhance on-field performance.

## 1. Introduction

Football (also known as soccer) is the most played and followed sport across the globe. It is indeed referred to as “the global game”. According to FIFA, there are about 300,000 clubs with 240 million registered players worldwide [1]. Soccer is also one of the most played activities among non-professional athletes across all age groups [2]. Furthermore, it is the most studied sport, with more than 14,000 articles and with an exponential growth from the 1970s [3].

### 1.1. Soccer Injuries

The dynamics of soccer matches and training increase the risk of injuries when compared with other kinds of sports. Considering the estimated cost of an injury in a top team professional player is about 500,000 USD per month [4].

Injury rates are significantly higher during soccer matches (10 times higher than the training injuries). A systematic review and meta-analysis published in 2020 by López-Valenciano et al. estimated an overall injury incidence of 9.6 injuries per 1000 h of match exposure [5]. Moreover, the Union of European Football Associations (UEFA) conducted a wide study on 23 soccer teams belonging to the 50 best European teams, showing an injury rate per player with an average of 2.0 injuries per season [6]. Moreover, there is an alarming incidence of re-injuries of 12% of all injuries, causing a significant period out-of-competition [6].

The location of injuries is set as follows: the highest rate occurs in the lower extremity; the other locations are the trunk and upper extremity. The lowest rate of injuries is located to neck and head. Regarding the lower extremity, the anatomic regions mostly involved are as follows in descending order: thigh, knee, ankle, hip, lower leg, and foot. In the same systematic review and meta-analysis, López-Valenciano et al. examined the different types of injuries occurring in soccer players, the most frequent type appeared to be muscle/tendon injuries, followed by contusions, joints and ligaments, fractures, and at last central/peripheral nervous system injuries [5]. In another systematic review and meta-analysis published in 2023, it was found that another type of injury, related to postural stress such that of low back pain, is consistently found in soccer players, with a frequency of 1165 events in a total of 48,577 injuries. It is interesting to see that the recurrence of lower back pain related to injuries is set in a percentage between 3% and 18.8% (with the longest follow-up set to 16 seasons in one of the studies analyzed). This also showed that the prevalence of lower back pain in soccer players is higher in female players than male players (respectively, 28% to 57% and 1% to 34%); these findings, as with those of other sports injuries (particularly anterior cruciate ligament and muscle injuries), have been more frequent during the post-COVID-19 era, where the rehabilitation could be considered as crucial [7,8,9,10,11].

The influence of injuries, e.g., ACL injuries, is highly impactful in players’ careers, with a decrease in the level of play 3 years later in 22.6% of the players and a total interruption of the professional career in 12.9% of them [12]. The impact of injuries is not only a burden for players, in the context of modern soccer, the economic side of the game is crucial. A recent retrospective study demonstrated, studying the seasons from 2014/2015 to 2020/2021, a one-rank drop for every 4.33 injured players in élite leagues, thus determining an average rank loss of 4.93 per season and a consequent financial loss of 24.2 million euros [13].

Moreover, despite the improvement of training techniques, the injury rate over one season, especially injuries concerning the hamstrings, increased significantly; this observation does not affect soccer matches, showing a stable injury rate, but has increased by a 4.0% per year rate for training injuries [14].

#### 1.1.1. Injury Prevention

Injury prevention is crucial to avoid injury and any long-term consequences for players that are out of competition for longer periods during a season. In this context, programs like FIFA 11+, can improve the training of professional footballers, giving the chance to reduce injury risk [4]. FIFA 11+ can improve performance by enhancing muscle strength, jump height, and sprint speed, it can likewise prevent injuries by increasing proprioceptive abilities, dynamic balance, and core stability [15]. Injury prevention must consider the perception of fatigue and asymmetry between agonist and antagonist contraction. To achieve this goal, it is crucial to evaluate athletes by periodically measuring muscular activation patterns as shown in kinematics and superficial electromyographic studies [16,17].

#### 1.1.2. Soccer Equipment

In this context, the equipment, along with training load, biomechanical profile, and personal history of injuries, could play a key role in terms of injury prevention. The soccer footwear needs to be personalized according to the athlete’s necessity to perform better and avoid injuries. In soccer, where the interface between the athlete and the playing surface is crucial for traction, stability, energy transfer, joint loading, and proprioception during high-intensity movements, the soccer footwear design must be structurally accurate [18]. The configuration of the studs, and their placement, are critical to joints (as knees and ankles) [19] and to the interface with the ground in terms of running, changing direction and other athletic gestures [20]. The soleplate could be crucially involved in comfort and plantar loading with implications for overuse injuries [21]. Today, upper materials are strongly bonded with functional aspects such as flexibility, breathability, ball control, and foot protection [22]. Another relevant part of the configuration of a soccer footwear depends on the type of playing surface, the mechanical properties of different turfs like artificial, natural grass, or hybrid pitches, affect the interaction with the footwear, impacting the structure of the footwear [18].

### 1.2. Biomechanics of Soccer Footwear

Each moment during a training or a soccer match depends on the interaction with the pitch. Running, cutting, sprinting, jumping, and kicking are tied with the soccer footwear, the ultimate interface between the athletic gesture and its execution [23]. Plantar pressure is one of the most crucial biomechanical aspects involved in soccer footwear. Elevated plantar loads, particularly under the heel and lateral forefoot, are associated with overuse injuries such as metatarsalgia, plantar fasciitis, and stress fractures [21,24]. Soleplate stiffness can modulate mid-foot load absorption. More rigid designs limit ankle dorsiflexion and increase lower limb stiffness, potentially heightening stress on mid-foot structures, especially in players with limited ankle dorsiflexion or high arches [20]. Another key factor is rotational traction: in soccer, many pivoting movements depend on resistance between the boot and the ground. Elevated torque and rotational stiffness, especially on artificial surfaces, can increase joint loading during cuts, potentially raising the risk of non-contact injuries such as ACL tears or ankle sprains [25,26]. Proprioception is essential for athletes to execute precise gestures, enhance balance and agility, and reduce lower-limb injury risk, as shown by proprioceptive training protocols that significantly improve soccer-specific technical performance [27]. The shoe upper should provide both lockdown and flexibility to increase the fine motor control without compromising comfort. Poor fit or excessive boot stiffness can reduce proprioception and neuromuscular responses while increasing the risk of imbalance and fatigue-related errors [28]. Overall, a mismatch between footwear design and a player’s biomechanical profile or playing condition may alter movement patterns and load distribution, potentially leading to compensatory strategies that increase injury risk. For example, soccer-specific single-leg tasks have been shown to reveal asymmetries and altered joint kinematics, such as increased knee valgus or reduced hip and ankle flexion, that are associated with non-contact lower limb injuries [29].

### 1.3. Aim of the Review

To the best of our knowledge, there is a lack of evidence on the impact of boot structure on injuries and performance, and no clear indication describing the single structures composing the modern soccer boots. This review aimed to provide a descriptive overview of the footwear in soccer structures, considering the modern manufacture, its personalization, and its impact on performance.

## 2. Materials and Methods

Initial research was undertaken to identify articles on the topic and to index terms for a full search strategy. Given the heterogeneity of the data and the difference between the various footwear structures, we decided to adopt a descriptive approach including more search strings. This narrative review involved 3 scientific databases, PubMed, Scopus, and Web of Science. We also selected additional articles from the bibliography of the included studies. The search strategy, as shown in Table 1, employed different search strings in relation to the soccer structure analysed. A broad and highly sensitive search strategy was intentionally employed to allow a comprehensive overview of the literature.

Two reviewers screened articles according to title and abstract, and then the full text of the articles was reviewed according to the aim of this review. In case of disagreement, consensus was reached with a third reviewer. The literature search was intended to provide a narrative overview with a structured but non-systematic approach. The eligibility criteria were defined to give a narrative and qualitative synthesis of the available literature. We included studies published from 1993 to 2025 that matched the following inclusion criteria: studies published in English, studies involving the role of soccer footwear in performance and injury prevention related to biomechanics and ground interface. We excluded studies written in any other language than English and studies which involved any other sport or any other equipment. The majority of the selected articles were observational. The papers were categorized by boot structure and main findings. Given the narrative nature of the review, data were summarized under thematic domains. No formal risk-of-bias or quantitative synthesis was conducted, consistent with narrative review methodology. The narrative approach was intended to capture contributions across different structures of the soccer boot without representing a fully reproducible format, thus giving a wide overview of this topic. This review did not involve human participants or new data collection; therefore, ethics committee approval was not required.

## 3. Boot Structure

### 3.1. Stud Design in Soccer Footwear, Functional Implications of Shape and Distribution

The design of studs (also named as cleats) is pivotal to shape the interaction between the athlete and the playing surface, they’re not just a traction-oriented feature but a biomechanical interface that influences performance, stability, and injury risk [18,19,20]. The geometry, the number, depth and distribution of the cleats determine the transmission of forces during the fundamentals of soccer, such as cutting, sprinting, pivoting, and so on [30,31,32]. Scientific literature emphasizes that stud characteristics must balance providing sufficient grip to enhance performance and avoiding excessive traction, to reduce injury risk, in particular rotational movements [18,25]. The complexity of the biomechanical impact of stud design requires a tailored approach in the selection of cleats’ configuration [33].

#### 3.1.1. Morphology and Surface Interaction

Stud morphology (shape, length, and geometry) has an influence during dynamic gestures on how the athlete’s foot interfaces with the ground, these features determine both the surface penetration and the stability and rotational freedom of the athlete [34]. Regarding the geometry of the studs, conical studs provide greater rotational release by allowing controlled pivoting and minimizing foot fixation. This configuration facilitates smoother transitions and reduces joint torque during rotational movements, especially at the knee and ankle joints, helping maintain physiological load distribution. In contrast, bladed or elongated studs maximize linear traction and sprint efficiency, but they increase resistance to rotational release. This “foot locking” effect significantly elevates internal joint torques during pivoting, increasing stress on the knee and heightening the risk of non-contact injuries, particularly ACL lesions [18,25,31,32,35,36]. Particularly, during cutting movements, the role of the cleats was analysed, showing a linear relationship between strain and axial force. This observation needs further studies to better understand this relationship [37] This distinction is critical in soccer, where the athletic gestures are characterized by quick changes of direction and rotational actions with excessive resistance to rotation, evidenced by higher torque peaks in the knee during pivoting on bladed soles, has been linked to elevated non-contact injury risk [18,25]; on the other hand, insufficient traction impairs agility and technical performance, especially on wet or natural grass, as highlighted in systematic reviews of cleat-surface interaction [32]. Surface types also affect the performance of the stud design. On natural grass, deeper penetration of the studs is required to gain a more consistent grip, while on artificial turf, longer studs may increase friction and shear forces to harmful levels [38,39]. Moreover, considering weather conditions, different kinds of climate can affect the shoe-surface interaction by changing the turf resistance, comparing May warm season grass to January cold season grass, there are notable differences in rotational traction [39,40]. Therefore, the morphology of the studs should be carefully selected according to playing surface conditions and adjusted to the player’s biomechanical profile, position, and style of play [32,34]. This tailored approach can optimize traction, agility, and rotational release while minimizing joint load and the risk of non-contact injuries [18,25,39]. Though no single configuration suits all scenarios, evidence suggests that surface-specific and role-adapted cleat designs may offer a safer and more efficient foot-ground interface [30,32].

#### 3.1.2. Stud Pattern Configuration

**Figure 1:**
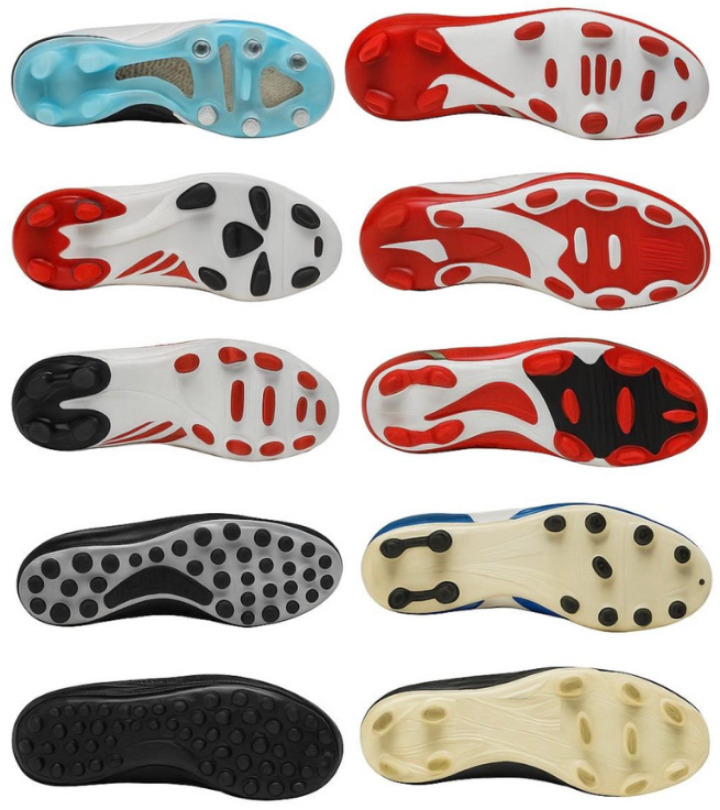
Stud structures (conical, bladed mixed) and stud distribution among various boots.

Beyond the individual morphology, the overall configuration and distribution of studs on the outsole have an important impact on the biomechanical function of the shoe itself. Stud placement, as shown in Figure 1, influences how the ground forces are transmitted through the foot and how the pressure is distributed across the plantar surface. This affects the behaviour of the foot through the gait cycle during the soccer-specific movements [33,41,42]. The configuration of the studs can be used to achieve specific functional outcomes; triangular or bladed studs positioned in the rearfoot increase shear resistance and braking efficiency, particularly during deceleration or lateral stabilization phases. This is attributed to their greater ground penetration and stability under load. In contrast, conical or semi-conical studs in the forefoot facilitate rapid propulsion and smoother directional changes, thanks to their lower rotational resistance and greater freedom of pivot, which are critical during agile manoeuvres and cutting tasks [34,42]. Studies employing pressure-mapping systems have shown that the density of stud patterns modulates plantar pressure zones. In particular, asymmetrical configuration may enhance pressure on the forefoot and midfoot during cutting or accelerating phases, potentially leading to stress-related injuries [38,43]. Excessive clustering of studs in specific areas of the outsole has been associated with altered joint kinematics, increased foot torsion, foot fixation, and impaired energy transfer efficiency. For instance, high stud density in the anterolateral forefoot region may elevate torsional stress during cutting manoeuvres, while clustering under the midfoot can hinder longitudinal foot flexibility and disrupt gait mechanics. Additionally, excessive rearfoot stud concentration can increase braking resistance, potentially transferring abnormal rotational loads to the knee and hip joints. These biomechanical implications are partially supported by plantar pressure and motion analyses from Lv et al. (2020) and Queen et al. (2008), which reveal how stud arrangement influences pressure distribution and joint loading during soccer-specific actions [34,42]. On the contrary, more evenly distributed patterns seem to support natural foot motion and reduce compensatory mechanics that may lead to fatigue or chronic injuries [38]. The choice of stud pattern should consider biomechanical aspects and not solely performance metrics. Studs should be aligned with the athlete’s foot to protect it from injuries and enhance the performance [38,44].

### 3.2. Upper Materials in Soccer Boots: Functional Roles and Biomechanical Implications

The upper of a soccer footwear is more than a structural envelope, it can be classified as an interface between the player and the ball itself, influencing ball control, comfort, proprioception and mechanical load distribution across the foot [22,25,45]. Moreover, the mechanical properties of the upper, such as its stiffness and compliance, can modulate the transmission of torsional forces during foot-ground interactions, potentially influencing injury risk and load distribution along the foot and ankle complex [25]. The choice of the correct soccer boot, in terms of dimensions and upper could also be crucial to players: proper fitting boots can enhance the interface between the player and the ground or the ball, thus improving the performance and the comfort during the game. On the other hand, the majority of the players have significantly higher arches, even if a direct correlation between the dimension of the boot and foot deformities wasn’t clearly observed [46].

In younger players, the foot dimension is also a matter in designing the correct shoe. Analysing the functional excess could be crucial and strictly depends on evaluating children’s foot measures. The correct dimension of the footwear, especially in children, reduces the possibility of developing foot abnormalities throughout growth, thus improving the comfort of foot contact, possibly enhancing the performance even in younger players [47,48].

To achieve the finest balance between foot comfort, player perceptions and injury prevention, the materials of the upper have significantly evolved from traditional leather to advanced synthetics with distinct functional properties [22].

Synthetic fibres are employed for enhanced durability, water resistance, and elasticity under stress, and their inherently stiffer architecture may improve lateral support and ball-strike precision during high-velocity movements. However, such rigidity can attenuate plantar tactile feedback, vibration sensitivity is known to decrease in soccer footwears compared with barefoot, and lead to increased pressure concentrations over bony prominences, as synthetics are often perceived as looser fitting and less anatomically conforming than traditional leathers [22,49,50]. Knitted uppers are commonly fabricated from elastic polymer yarns and engineered in flat or 3D knit structures to provide a sock-like fit that conforms dynamically to the foot, enhancing proprioception, comfort, and freedom of movement. Although generally less durable than synthetic leather, knitted uppers offer superior breathability, moisture management, and ergonomic conformity, with flat- or 3D-knits demonstrating more precise foot wrapping and mechanical adaptation during dynamic tasks, making them ideal for athletes requiring high foot dexterity [51,52,53]. Engineered mesh materials combine lightweight structures with localized reinforcements; such uppers enhance breathability and reduce thermal stress (including lower in-shoe temperature and humidity rise), thereby improving comfort during prolonged matches. In soccer-specific simulations, reduced foot discomfort has been linked to lower heart rates in the final 30 min and better maintenance of jump power, underscoring the link between upper comfort and sustained performance [49,54]. Furthermore, the zonal design of engineered mesh uppers allows differentiated stiffness across the forefoot, midfoot, and heel, made possible by integrating denser mesh or polymer reinforcements in specific areas, thereby optimizing dynamic foot behaviour and stress distribution during multiplantar tasks [55]. Biomechanically, it is important to emphasize how dorsal pressure distribution contributes to foot stability. Stiffer uppers can enhance shooting precision and lateral stability by minimizing upper deformation and improving energy transfer during ball contact, however, excessive rigidity may lead to increased localized pressure over the foot dorsum, potentially reducing comfort and elevating the risk of overuse injuries. Therefore, an optimal balance between stiffness and compliance must be achieved to support performance while minimizing mechanical stress [56,57,58].

#### Hybrid Constructions: Functional Integration of Upper Materials

Using single materials can be limiting for their various features, by this means, modern soccer footwears are increasingly adopting hybrid upper constructions to optimize performance and guarantee protection from injuries and athletes’ safety [41,59]. A common strategy involves using reinforced synthetic microfibers or PU films that can absorb up to 30% more energy than EVA/TPU and reduce peak forces transmitted during impact by up to 36.9%, properties that, when used as localized PU films in upper structures, can enhance impact resistance and accuracy during ball striking in high stress areas like toe box, lateral forefoot and instep to increase impact resistance and ball striking precision [60,61]. The stiffer components should be integrated with knit or mesh zones over the midfoot and the ankle collar to enhance comfort, adaptability, and proprioceptive sensitivity furthermore increasing freedom of movement [62,63]. New advancements in lamination and heat-bonding techniques enable seamless integration of dissimilar materials, minimizing areas that might create pressure points or friction. These technologies allow footwear to be tailored not only by player position but also by foot morphology, which can be mapped precisely with new 4D models and an athlete’s clinical injury history, introducing a performance-driven, customized design approach [64,65].

### 3.3. Soleplate Design and Load Distribution

The soleplate plays a pivotal role in mediating the distribution of the mechanical forces during the athlete’s movements, the structural design is often underestimated despite its critical impact on performance and injury prevention [21]. An optimal soleplate should optimize the load distribution across the plantar surface during dynamic activities. Devices like pressure insoles and robotic simulators demonstrated that the soleplate construction and design affect the magnitude and timing of peak plantar loads [21,49].

#### 3.3.1. Structural Properties of the Soleplate

**Figure 2:**
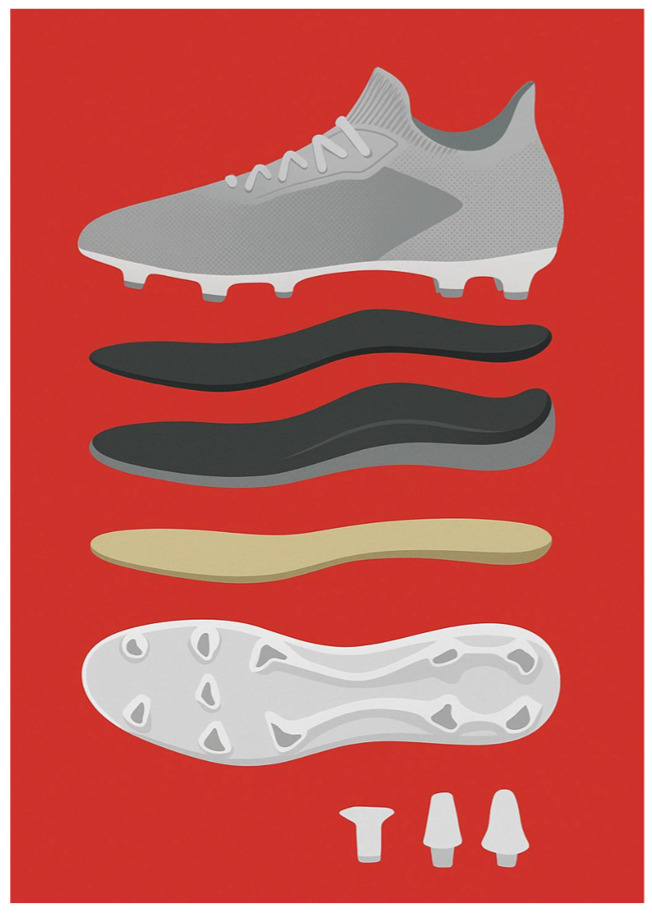
Different soleplates in terms of thickness and designs.

Soleplate flexibility could affect proprioception and neuromuscular control; increased forefoot stiffness may improve sprinting and performance, especially in forwards or wingers, on the other hand, it may compromise multidirectional tasks typical of midfielders [66,67]. Designs of the soleplate (Figure 2) that incorporate more flexible structures have a more adaptive shape to the foot and better adapt to the characteristics of the ground [68]. The interaction between the soleplate and playing surface needs to be analysed. On natural grass, soleplates with moderate torsional stiffness seem to offer the best compromise between stability and rotational freedom [69,70]. Modern soleplates are typically designed in materials like thermoplastic polyurethane (TPU), nylon composites, or carbon fibre reinforcements. Each material has distinct profiles that modulate energy return, comfort, and force transmission to the foot [60,68,71]. Analysing drop tests can give information regarding shock absorption, particularly Poron insoles, which may provide better impact attenuation than Poron/gel structures [21].

#### 3.3.2. Load Distribution and Shock Absorption

**Figure 3:**
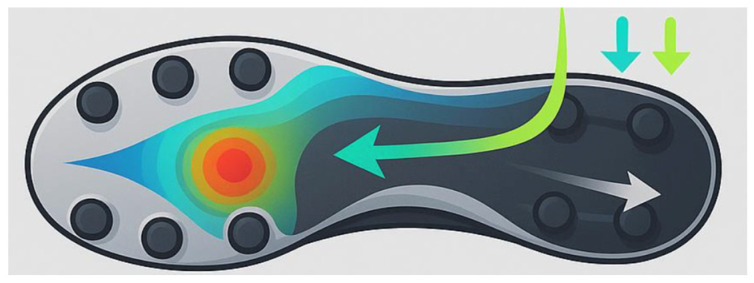
Different possible distribution of the ground forces on the interface between the soccer boot and the ground.

During soccer-specific movements, the foot is often subjected to rapidly changing forces (Figure 3); if these forces are not distributed across the plantar surface, they can generate focal peaks that increase stress-related injuries, particularly over the metatarsal heads, heel, and midfoot [24,72,73]. Plantar pressure mapping studies reveal that soleplates that integrate flexible zones or adaptive cushioning systems can reduce localized forefoot loading, and shock absorption capabilities are linked to the reduction of stress reactions and overload injuries [21]. In artificial turf or hybrid grounds, a more adaptive soleplate is required to reduce the shear stress given by the ground [32,74]. Soccer footwear is known to increase plantar pressure in some specific foot areas like the rearfoot and midfoot. The highest pressure is often described in M1 (first metatarsal head), showing how it’s crucial to integrate statistical analysis integrated with personal variability in order to design a good soleplate [75]. In this context, it’s important to emphasize that there’s not a static solution for the design of the soleplate, thus, it needs to be tailored on individual biomechanics and surface conditions. Integrating pressure-mapping technologies and player-specific data will be crucial to identify the best possible solution for the athlete [76,77].

## 4. Clinical Implications

### 4.1. Rotational Resistance of Boot Studs and Injury Risk

One of the most important biomechanical aspects of soccer footwear design is managing rotational resistance, the frictional force that resists pivoting or twisting movements between the footwear and the playing surface. While a certain degree of resistance is necessary for stability and performance, excessive rotational grip can increase the risk of non-contact injuries to the ankle and joints [25,70]. Higher rotational tractions are directly associated with elevated torque transmission to the lower limbs during turning and cutting actions, and artificial turf surfaces amplify this risk. Studs that penetrate deeply into the ground can inhibit natural rotational release and increase the mechanical load on ligaments during rapid decelerations or direction changes [32,70]. On the other side, a recent retrospective study showed that, in female players, the risk of anterior cruciate ligament tears was significantly higher in those who wore conical studded shoes, showing a discrepancy with the previous literature, which focused on male players, highlighting the necessity of further studies to better assess the difference between female players and male players [78]. This evidence shows the importance of finding a balance between the need to increase performance and, on the other hand, reducing the risk of non-contact injuries. In this context, studies are increasingly focusing on reducing unwanted torsional resistance by using rounded edges, dual-density studs, and, above all, hybrid configurations to allow a safer disengagement during pivotal movements [79,80,81]. Gaining a better knowledge of the biomechanical dynamics of torsional resistance during soccer-related movements could be crucial to define the correct pattern and reduce injuries; in this context, the analysis of Ground Reaction Forces by using instrumented soccer boots could play a pivotal role. The studs of this experimental shoe are equipped with a sensor that measures any kind of deformation during matches [82]. Overall, injury risk is strictly associated with cleat configuration, after dividing boots into very aggressive (bladed studs), mildly aggressive (hybrid configurations), and nonaggressive (conical studs). A cohort study conducted on injured players in the English Premier League showed that while nonaggressive patterns showed no significantly higher or lower odds of injury, very aggressive patterns increased the risk, and mildly aggressive patterns appeared to be safer. In particular, very aggressive stud patterns showed higher odds concerning ankle and knee injuries, and nonaggressive stud patterns showed higher odds concerning hamstrings injuries [83].

### 4.2. Customization of Boot Studs

The concept of functional footwear matching emphasizes the alignment of stud characteristics with contextual and physiological variables. Soft grass requires longer and more penetrative studs to enhance traction and stability; artificial turf requires shorter and rounder configurations to reduce shear stress [39,44,84]. Similarly, attacking players benefit of asymmetrical stud patterns that prioritize propulsion, while defending players may benefit from denser heel arrangements for braking and lateral control [38,85]. Individual factors like foot morphology, injury history, and neuromuscular profile should guide the selection process: athletes who suffered from ankle sprains should require configurations that improve medial-lateral control, with a higher density of studs on the sides [44]. While, those recovering from ACL reconstruction may benefit from reduced torsional resistance, with conical studs or dual density studs, to protect healing ligaments [86].

### 4.3. Fatigue, Proprioception, and Overuse Risk Linked to Soleplate Design

The soleplate and insole structure play a critical role in modulating sensory feedback and neuromuscular control. Proprioceptive stimulation from the plantar surface can enhance postural stability and delay neuromuscular fatigue during prolonged efforts, potentially reducing injury risk [87,88]. An altered foot-ground interaction mediated by the soleplate can lead to biomechanical compensation patterns that increase neuromuscular fatigue over time and reduce postural control [89]. To mitigate this risk, it’s important to design a player-specific tuning of soleplate stiffness and proprioceptive responsiveness [21,87,90,91]. Moreover, plantar pressures, need to be analysed to better understand the injury risk in athletes who are involved in sprinting movements and side cuts; as mentioned before fatigue can be a critical aspect in designing a soleplate, in fact, it appears substantially during sprints [92].

#### Functional Customization

Translating biomechanical insights into practical footwear choices remains a key challenge. Soleplates with enhanced forefoot stiffness may be advantageous for forwards seeking maximal push-off power; on the other hand, defenders may benefit from a higher stability guaranteed by wider torsional bars or stiffer shanks. For both kinds of players, excessive rigidity could impair dynamic balance, especially on softer grounds like natural grass [39,69,83]. Moreover, modular soleplate technologies, which can partially adjust stiffness or traction zones, are gaining the ability to adapt to individual anatomical and performance profiles [93].

## 5. Conclusions

By the present comprehensive narrative review, we showed how modern soccer footwear should not be considered only as a piece of sportswear, but also as a biomechanical interface between the player and the ground. Indeed, the different components, upper, insole, outsole, cleats, modulate load distribution, proprioceptive perceptions, and comfort during soccer-specific tasks. All these components could modify the performance of the player and the risk of non-contact injuries. We also showed how different grounds need different structures, in a world in which the technologies of the playing surface are improving day by day. But standardization isn’t an option; different roles (attack, defence, midfield) need different cleat patterns and show different plantar pressures. While these considerations are already known between the players and the teams, there’s still a lack of awareness about how different biomechanical profiles can affect the choice of the right soccer boot.

This paper comes with inherent limitations. The narrative design of this review, whether giving a wide overview of the available research, limits the level of evidence. Moreover, the quantitative analysis was not possible due to the design of this review, thus we only depicted a qualitative synthesis.

Therefore, a key outcome of this review is the clear need for personalization in terms of footwear. Players with different anatomical features and foot morphologies, injury histories, and neuromuscular profiles require different soleplate stiffness, traction profiles, and upper responsiveness to optimize performance and safety. Emerging technologies such as modular soleplates, pressure mapping systems, and adaptive materials represent promising frontiers in performance-specific and injury-preventive footwear design.

Future research should focus on integrating player-specific biomechanical data with in-field performance metrics to validate and refine these approaches. Further studies, with more structured protocols should focus on structuring a personalized approach, considering anatomical differences among the athlete’s foot. In doing so, the soccer footwear may evolve from standardized equipment to a truly individualized extension of the athlete’s body, capable of enhancing performance while minimizing injury risk.

## Figures and Tables

**Table 1:** Search Strategy.

Biomechanics of footwear	((“football” OR “soccer”) AND (“shoe” OR “shoes” OR “boot” OR “boots” OR “cleat” OR “cleats”) AND (“biomechanics” OR “kinematics” OR “kinetics” OR “motion analysis”))
Studs	((“boots” OR “shoes” OR “shoe” OR “boot”) AND (“stud” OR “studs” OR “studded” OR “cleat” OR “cleats”) AND (“football” OR “soccer”))
Uppers	(“Soccer” [Mesh] OR football OR “soccer players”) AND (“Footwear uppers” OR upper material OR upper construction OR “shoe upper” OR “soccer shoe upper” OR “football boot upper”) AND (performance OR comfort OR biomechanics OR shooting OR perception)
Insole	((“football” OR “soccer”) AND (“shoe” OR “shoes” OR “boot” OR “boots” OR “cleat” OR “cleats”) AND (“insole” OR “soleplate” OR “soleplate design” OR “insole design”))

## Data Availability

No new data were created or analyzed in this study. Data sharing is not applicable to this article.
